# Cs_2_UPd_3_Se_6_

**DOI:** 10.1107/S1600536810053924

**Published:** 2011-01-12

**Authors:** George N. Oh, James A. Ibers

**Affiliations:** a Department of Chemistry, Northwestern University, 2145 Sheridan Rd., Evanston, IL 60208-3113, USA

## Abstract

Dicaesium uranium(IV) tripalladium(II) hexa­selenide, Cs_2_UPd_3_Se_6_, crystallizes in the space group *Fmmm* in the Ba_2_NaCu_3_O_6_ structure type. The asymmetric unit comprises the following atoms with site symmetries as shown: U1 (*mm*2), Cs1 (222), Cs2 (*m*2*m*), Pd1 (*.m.*), Pd2 (2*mm*), Se1 (*m..*), and Se2 (1). This layered structure contains six edge-sharing square-planar [PdSe_4_] units that form a hexa­gon. These, in turn, edge-share with [USe_6_] trigonal–prismatic units, forming an extended layer parallel to (010). The layers are stacked along [010]. They are staggered, and are separated by the Cs atoms. The Cs atoms are either coordinated in a square anti­prism of Se atoms or are ten-coordinate, with one square face and the opposite face hexa­gonal.

## Related literature

Ba_2_NaCu_3_O_6_ was reported by Tams & Müller-Buschbaum (1992 ▶). For related structures, see: Daoudi & Noël (1989 ▶); Bronger *et al.* (1991 ▶); Yao & Ibers (2008 ▶); Huang *et al.* (2001 ▶); Klepp *et al.* (1996 ▶). For synthetic details, see: Bugaris & Ibers (2008 ▶); Haneveld & Jellinek (1969 ▶). For computational details, see: Gelato & Parthé (1987 ▶); Le Page (1988 ▶).

## Experimental

### 

#### Crystal data

Cs_2_UPd_3_Se_6_
*M* *_r_* = 1296.81
Orthorhombic, 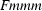

*a* = 10.1034 (5) Å
*b* = 15.5046 (8) Å
*c* = 17.5503 (8) Å
*V* = 2749.2 (2) Å^3^
*Z* = 8
Mo *K*α radiation
μ = 36.67 mm^−1^
*T* = 100 K
0.21 × 0.17 × 0.01 mm

#### Data collection

Bruker APEXII CCD diffractometer
Absorption correction: numerical (face indexed using *SADABS*; Sheldrick, 2008*b* ▶) *T*_min_ = 0.049, *T*_max_ = 0.689
8099 measured reflections
963 independent reflections
921 reflections with *I* > 2σ(*I*)
*R*_int_ = 0.035

#### Refinement

*R*[*F* ^2^ > 2σ(*F* ^2^)] = 0.019
*wR*(*F* ^2^) = 0.055
*S* = 1.46
963 reflections
38 parameters
Δρ_max_ = 1.34 e Å^−3^
Δρ_min_ = −2.21 e Å^−3^

### 

Data collection: *APEX2* (Bruker, 2009 ▶); cell refinement: *SAINT* (Bruker, 2009 ▶); data reduction: *SAINT*; program(s) used to solve structure: *SHELXS97* (Sheldrick, 2008*a* ▶); program(s) used to refine structure: *SHELXL97* (Sheldrick, 2008*a* ▶); molecular graphics: *CrystalMaker* (Palmer, 2009 ▶); software used to prepare material for publication: *SHELXL97*.

## Supplementary Material

Crystal structure: contains datablocks I, global. DOI: 10.1107/S1600536810053924/wm2437sup1.cif

Structure factors: contains datablocks I. DOI: 10.1107/S1600536810053924/wm2437Isup2.hkl

Additional supplementary materials:  crystallographic information; 3D view; checkCIF report

## Figures and Tables

**Fig. 1.**
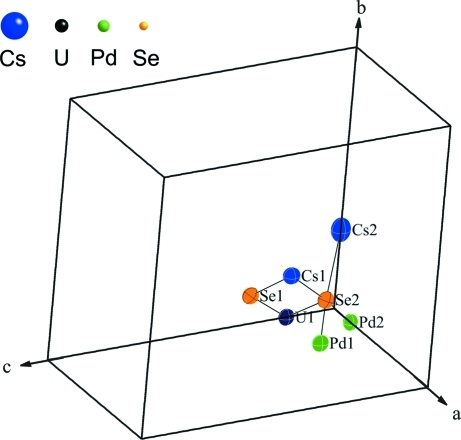
: Asymmetric unit of Cs2UPd3Se6 (95% probability ellipsoids).

**Fig. 2.**
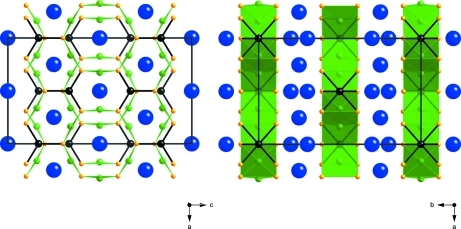
: Structure of Cs2UPd3Se6viewed down the b-axis (left) and the c-axis (right).

**Fig. 3.**
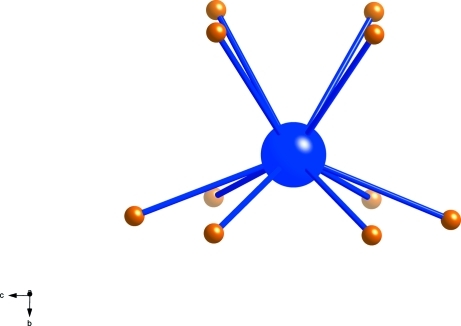
: Coordination environment of atom Cs2.

## supplementary crystallographic information

### Comment

As part of continuing efforts to synthesize new uranium chalcogenide compounds, Cs_2_UPd_3_Se_6_ was synthesized. It crystallizes in the orthorhombic space group *Fmmm* in the Ba_2_NaCu_3_O_6_ structure type (Tams & Müller-Buschbaum, 1992). The asymmetric unit comprises the following atoms with site symmetries as shown: U1 (*mm*2); Cs1 (222); Cs2 (*m*2*m*); Pd1 (*.m.*); Pd2 (2*mm*); Se1 (*m..*); and Se2 (1) (Fig. 1). This layered structure (Fig. 2) contains six edge-sharing square-planar [PdSe_4_] units that form a hexagon. These, in turn, edge-share with [USe_6_] trigonal-prism units to form an extended layer parallel to (010). The layers are stacked along [010] and are separated by Cs atoms. The Cs atoms are either coordinated in a square antiprism of Se atoms or are ten-coordinate, with one square face and the opposite face hexagonal (Fig. 3).

The shortest Se–Se distance is 3.3344 (9) Å, far longer than a single bond. Thus, formal oxidation states may be assigned as follows: Cs: +1; U: +4; Pd: +2; Se: -2. Pd^2+^ typically has a square-planar coordination. In contrast, trigonal-prismatic coordination is unusual for U^4+^, though it is known, for example in Ba_4_Cr_2_US_9_ (Yao & Ibers, 2008).

U–Se distances are 2.8353 (5) Å and 2.8704 (7) Å, similar to those in other compounds containing six-coordinate U^4+^ atoms, such as in CsUCuSe_3_ (Huang *et al.*, 2001), where the U–Se distances range from 2.8265 (6) Å to 2.8611 (8) Å. Pd–Se distances are typical for square-planar Pd^2+^ atoms, ranging from 2.4516 (5) Å to 2.4736 (6) Å, similar to those of 2.453 (6) Å to 2.456 (7) Å in Cs_2_Pd_3_Se_4_ (Bronger *et al.*, 1991) and those of 2.476 (1) Å and 2.479 (1) Å found in UPdSe_3_ (Daoudi & Noël, 1989). The Cs1–Se distances, which are 3.4653 (5) Å and 3.4682 (5) Å, are shorter than are typical for eight-coordinate Cs. Typical are those in CsUCuSe_3_ which range from 3.5825 (16) Å to 3.8246 (11) Å. On the other hand, the ten-coordinate Cs2—Se distances are typical. They range from 3.7847 (7) Å to 3.9511 (7) Å, to be compared to 3.660 (3) Å to 3.961 (7) Å in CsFe_2_Se_3_ (Klepp *et al.*, 1996).

### Experimental

Black hexagonal plates of Cs_2_UPd_3_Se_6_ were synthesized by the combination of U (0.063 mmol), Pd (Johnson Matthey 99.94%, 0.063 mmol), Se (Cerac 99.999%, 0.253 mmol), and 125 mg CsCl (Aldrich 99.9%, 0.743 mmol) as a flux. U filings (Oak Ridge National Laboratory) were powdered by hydridization and subsequent decomposition under heat and vacuum (Bugaris & Ibers, 2008), in a modification of a previous literature method (Haneveld & Jellinek, 1969). The mixture was loaded into a carbon-coated fused-silica tube in an Ar filled glove box and then sealed under 10 ^-4^ Torr vacuum. The reaction was heated to 1273 K in 48 h, held there for 6 h, cooled to 1223 K in 12 h, held there for 24 h, then cooled to 298 K at 3.2 K/h.

### Refinement

The structure was standardized by means of the program *STRUCTURE TIDY* (Gelato & Parthé, 1987). The highest peak in the difference Fourier map of 1.3 (3) e/Å^3^ is 1.24 Å from atom U1 and the deepest hole of -2.2 (3) e/Å^3^ is 0.84Å from atom U1. The program *ADDSYM* (Le Page, 1988) was used to confirm that no symmetry was missed.

### Crystal data

**Table:**

Cs_2_UPd_3_Se_6_	*F*(000) = 4352
*M**_r_* = 1296.81	*D*_x_ = 6.266 Mg m^−^^3^
Orthorhombic, *F**m**m**m*	Mo *K*α radiation, λ = 0.71073 Å
Hall symbol: -F 2 2	Cell parameters from 5598 reflections
*a* = 10.1034 (5) Å	θ = 2.3–28.2°
*b* = 15.5046 (8) Å	µ = 36.67 mm^−^^1^
*c* = 17.5503 (8) Å	*T* = 100 K
*V* = 2749.2 (2) Å^3^	Hexagonal plate, black
*Z* = 8	0.21 × 0.17 × 0.01 mm

### Data collection

**Table:**

Bruker APEXII CCD diffractometer	963 independent reflections
Radiation source: fine-focus sealed tube	921 reflections with *I* > 2σ(*I*)
graphite	*R*_int_ = 0.035
ω scans	θ_max_ = 28.6°, θ_min_ = 2.3°
Absorption correction: numerical (face indexed using *SADABS*; Sheldrick, 2008*b*)	*h* = −13→13
*T*_min_ = 0.049, *T*_max_ = 0.689	*k* = −20→20
8099 measured reflections	*l* = −23→23

### Refinement

**Table:**

Refinement on *F*^2^	Primary atom site location: structure-invariant direct methods
Least-squares matrix: full	Secondary atom site location: difference Fourier map
*R*[*F*^2^ > 2σ(*F*^2^)] = 0.019	[1.00000 + 0.00000exp(0.00(sinθ/λ)^2^)]/ [σ^2^(*F*_o_^2^) + 0.0000 + 0.0000**P* + (0.0241*P*)^2^ + 0.0000sinθ/λ] where *P* = 1.00000*F*_o_^2^ + 0.00000*F*_c_^2^
*wR*(*F*^2^) = 0.055	(Δ/σ)_max_ < 0.001
*S* = 1.46	Δρ_max_ = 1.34 e Å^−^^3^
963 reflections	Δρ_min_ = −2.21 e Å^−^^3^
38 parameters	Extinction correction: *SHELXL97* (Sheldrick, 2008*a*), Fc^*^=kFc[1+0.001xFc^2^λ^3^/sin(2θ)]^-1/4^
0 restraints	Extinction coefficient: 0.000083 (9)

### Fractional atomic coordinates and isotropic or equivalent isotropic displacement parameters (Å^2^)

**Table:**

	*x*	*y*	*z*	*U*_iso_*/*U*_eq_	
U1	0.0000	0.0000	0.166826 (18)	0.00859 (12)	
Cs1	0.2500	0.2500	0.2500	0.00986 (14)	
Cs2	0.0000	0.30128 (4)	0.0000	0.01609 (16)	
Pd1	0.33694 (4)	0.0000	0.15828 (3)	0.00866 (14)	
Pd2	0.17351 (6)	0.0000	0.0000	0.00843 (16)	
Se1	0.0000	0.10753 (4)	0.29996 (3)	0.00912 (15)	
Se2	0.19159 (4)	0.11000 (3)	0.09981 (2)	0.00908 (13)	

### Atomic displacement parameters (Å^2^)

**Table:**

	*U*^11^	*U*^22^	*U*^33^	*U*^12^	*U*^13^	*U*^23^
U1	0.00523 (18)	0.0108 (2)	0.00978 (19)	0.000	0.000	0.000
Cs1	0.0078 (2)	0.0105 (3)	0.0113 (3)	0.000	0.000	0.000
Cs2	0.0104 (3)	0.0233 (4)	0.0146 (3)	0.000	0.000	0.000
Pd1	0.0049 (2)	0.0109 (3)	0.0102 (3)	0.000	−0.00063 (15)	0.000
Pd2	0.0067 (3)	0.0106 (4)	0.0080 (3)	0.000	0.000	0.000
Se1	0.0058 (3)	0.0107 (3)	0.0109 (3)	0.000	0.000	0.0004 (2)
Se2	0.0065 (2)	0.0105 (3)	0.0103 (2)	−0.00038 (17)	−0.00027 (15)	−0.00014 (16)

### Geometric parameters (Å, °)

**Table:**

U1—Se2	2.8353 (5)	Cs2—Se2^xvi^	3.8301 (5)
U1—Se2^i^	2.8353 (5)	Cs2—Se2	3.9511 (7)
U1—Se2^ii^	2.8353 (5)	Cs2—Se2^xvii^	3.9511 (7)
U1—Se2^iii^	2.8353 (5)	Cs2—Se2^ii^	3.9511 (7)
U1—Se1^i^	2.8704 (7)	Cs2—Se2^xviii^	3.9511 (7)
U1—Se1	2.8704 (7)	Cs2—Pd1^ix^	4.4635 (6)
U1—Pd1^i^	3.4076 (4)	Cs2—Pd1^xix^	4.4635 (6)
U1—Pd1	3.4076 (4)	Pd1—Se1^xi^	2.4557 (6)
U1—Pd2^iv^	3.4125 (4)	Pd1—Se1^v^	2.4557 (6)
U1—Pd2	3.4125 (4)	Pd1—Se2^iii^	2.4736 (6)
U1—Pd1^v^	3.4836 (6)	Pd1—Se2	2.4736 (6)
U1—Pd1^vi^	3.4836 (6)	Pd1—Pd2	3.2316 (6)
Cs1—Se2^vii^	3.4653 (5)	Pd1—U1^v^	3.4836 (6)
Cs1—Se2^viii^	3.4653 (5)	Pd1—Cs1^v^	4.2880 (3)
Cs1—Se2^ix^	3.4653 (5)	Pd1—Cs2^xx^	4.4636 (6)
Cs1—Se2	3.4653 (5)	Pd1—Cs2^xv^	4.4636 (6)
Cs1—Se1	3.4682 (5)	Pd2—Se2	2.4516 (5)
Cs1—Se1^ix^	3.4682 (5)	Pd2—Se2^xxi^	2.4516 (5)
Cs1—Se1^x^	3.4682 (5)	Pd2—Se2^iii^	2.4516 (5)
Cs1—Se1^xi^	3.4682 (5)	Pd2—Se2^xviii^	2.4516 (5)
Cs1—Pd1	4.2880 (3)	Pd2—Pd1^xviii^	3.2316 (6)
Cs1—Pd1^xii^	4.2881 (3)	Pd2—U1^iv^	3.4125 (4)
Cs1—Pd1^v^	4.2881 (3)	Pd2—Cs2^xv^	4.5137 (6)
Cs1—Pd1^ix^	4.2881 (3)	Pd2—Cs2^xx^	4.5137 (6)
Cs2—Se1^x^	3.7847 (7)	Se1—Pd1^vi^	2.4557 (6)
Cs2—Se1^xiii^	3.7847 (7)	Se1—Pd1^v^	2.4557 (6)
Cs2—Se2^ix^	3.8301 (5)	Se1—Cs1^x^	3.4682 (5)
Cs2—Se2^xiv^	3.8301 (5)	Se1—Cs2^x^	3.7847 (7)
Cs2—Se2^xv^	3.8301 (5)	Se2—Cs2^xv^	3.8301 (5)
			
Se2—U1—Se2^i^	130.98 (2)	Se2^xv^—Cs2—Se2^ii^	150.563 (16)
Se2—U1—Se2^ii^	86.111 (19)	Se2^xvi^—Cs2—Se2^ii^	70.624 (13)
Se2^i^—U1—Se2^ii^	73.96 (2)	Se2—Cs2—Se2^ii^	58.670 (15)
Se2—U1—Se2^iii^	73.96 (2)	Se2^xvii^—Cs2—Se2^ii^	52.634 (14)
Se2^i^—U1—Se2^iii^	86.111 (19)	Se1^x^—Cs2—Se2^xviii^	133.761 (13)
Se2^ii^—U1—Se2^iii^	130.98 (2)	Se1^xiii^—Cs2—Se2^xviii^	82.482 (12)
Se2—U1—Se1^i^	133.399 (11)	Se2^ix^—Cs2—Se2^xviii^	94.230 (11)
Se2^i^—U1—Se1^i^	89.330 (14)	Se2^xiv^—Cs2—Se2^xviii^	117.735 (13)
Se2^ii^—U1—Se1^i^	133.398 (11)	Se2^xv^—Cs2—Se2^xviii^	70.624 (13)
Se2^iii^—U1—Se1^i^	89.330 (14)	Se2^xvi^—Cs2—Se2^xviii^	150.563 (16)
Se2—U1—Se1	89.331 (14)	Se2—Cs2—Se2^xviii^	52.633 (14)
Se2^i^—U1—Se1	133.398 (11)	Se2^xvii^—Cs2—Se2^xviii^	58.670 (15)
Se2^ii^—U1—Se1	89.330 (14)	Se2^ii^—Cs2—Se2^xviii^	82.711 (18)
Se2^iii^—U1—Se1	133.398 (11)	Se1^x^—Cs2—Pd1^ix^	33.369 (10)
Se1^i^—U1—Se1	71.02 (3)	Se1^xiii^—Cs2—Pd1^ix^	108.628 (18)
Se2—U1—Pd1^i^	131.589 (12)	Se2^ix^—Cs2—Pd1^ix^	33.614 (9)
Se2^i^—U1—Pd1^i^	45.548 (10)	Se2^xiv^—Cs2—Pd1^ix^	109.694 (16)
Se2^ii^—U1—Pd1^i^	45.548 (10)	Se2^xv^—Cs2—Pd1^ix^	74.720 (12)
Se2^iii^—U1—Pd1^i^	131.589 (12)	Se2^xvi^—Cs2—Pd1^ix^	76.571 (12)
Se1^i^—U1—Pd1^i^	92.053 (8)	Se2—Cs2—Pd1^ix^	93.521 (9)
Se1—U1—Pd1^i^	92.053 (8)	Se2^xvii^—Cs2—Pd1^ix^	167.125 (9)
Se2—U1—Pd1	45.549 (10)	Se2^ii^—Cs2—Pd1^ix^	115.027 (9)
Se2^i^—U1—Pd1	131.590 (12)	Se2^xviii^—Cs2—Pd1^ix^	127.822 (8)
Se2^ii^—U1—Pd1	131.590 (12)	Se1^x^—Cs2—Pd1^xix^	33.369 (10)
Se2^iii^—U1—Pd1	45.549 (10)	Se1^xiii^—Cs2—Pd1^xix^	108.628 (18)
Se1^i^—U1—Pd1	92.053 (8)	Se2^ix^—Cs2—Pd1^xix^	76.571 (12)
Se1—U1—Pd1	92.052 (8)	Se2^xiv^—Cs2—Pd1^xix^	74.720 (12)
Pd1^i^—U1—Pd1	174.96 (2)	Se2^xv^—Cs2—Pd1^xix^	109.694 (16)
Se2—U1—Pd2^iv^	89.701 (13)	Se2^xvi^—Cs2—Pd1^xix^	33.614 (9)
Se2^i^—U1—Pd2^iv^	45.040 (11)	Se2—Cs2—Pd1^xix^	115.028 (9)
Se2^ii^—U1—Pd2^iv^	45.040 (11)	Se2^xvii^—Cs2—Pd1^xix^	127.822 (8)
Se2^iii^—U1—Pd2^iv^	89.702 (13)	Se2^ii^—Cs2—Pd1^xix^	93.520 (9)
Se1^i^—U1—Pd2^iv^	134.300 (11)	Se2^xviii^—Cs2—Pd1^xix^	167.125 (9)
Se1—U1—Pd2^iv^	134.300 (11)	Pd1^ix^—Cs2—Pd1^xix^	43.318 (11)
Pd1^i^—U1—Pd2^iv^	56.567 (13)	Se1^xi^—Pd1—Se1^v^	85.52 (3)
Pd1—U1—Pd2^iv^	118.389 (14)	Se1^xi^—Pd1—Se2^iii^	171.97 (3)
Se2—U1—Pd2	45.038 (11)	Se1^v^—Pd1—Se2^iii^	93.095 (17)
Se2^i^—U1—Pd2	89.702 (13)	Se1^xi^—Pd1—Se2	93.096 (18)
Se2^ii^—U1—Pd2	89.702 (13)	Se1^v^—Pd1—Se2	171.96 (3)
Se2^iii^—U1—Pd2	45.040 (11)	Se2^iii^—Pd1—Se2	87.18 (3)
Se1^i^—U1—Pd2	134.300 (11)	Se1^xi^—Pd1—Pd2	126.819 (19)
Se1—U1—Pd2	134.300 (11)	Se1^v^—Pd1—Pd2	126.82 (2)
Pd1^i^—U1—Pd2	118.388 (14)	Se2^iii^—Pd1—Pd2	48.701 (14)
Pd1—U1—Pd2	56.568 (13)	Se2—Pd1—Pd2	48.699 (14)
Pd2^iv^—U1—Pd2	61.821 (18)	Se1^xi^—Pd1—U1	131.077 (15)
Se2—U1—Pd1^v^	92.446 (11)	Se1^v^—Pd1—U1	131.077 (15)
Se2^i^—U1—Pd1^v^	133.501 (12)	Se2^iii^—Pd1—U1	54.909 (13)
Se2^ii^—U1—Pd1^v^	133.501 (12)	Se2—Pd1—U1	54.909 (13)
Se2^iii^—U1—Pd1^v^	92.445 (11)	Pd2—Pd1—U1	61.793 (13)
Se1^i^—U1—Pd1^v^	44.171 (11)	Se1^xi^—Pd1—U1^v^	54.538 (17)
Se1—U1—Pd1^v^	44.172 (11)	Se1^v^—Pd1—U1^v^	54.538 (17)
Pd1^i^—U1—Pd1^v^	120.746 (11)	Se2^iii^—Pd1—U1^v^	130.265 (15)
Pd1—U1—Pd1^v^	64.299 (14)	Se2—Pd1—U1^v^	130.266 (15)
Pd2^iv^—U1—Pd1^v^	177.312 (13)	Pd2—Pd1—U1^v^	177.495 (17)
Pd2—U1—Pd1^v^	120.866 (11)	U1—Pd1—U1^v^	115.701 (14)
Se2—U1—Pd1^vi^	133.502 (12)	Se1^xi^—Pd1—Cs1	53.965 (11)
Se2^i^—U1—Pd1^vi^	92.445 (11)	Se1^v^—Pd1—Cs1	129.73 (2)
Se2^ii^—U1—Pd1^vi^	92.445 (11)	Se2^iii^—Pd1—Cs1	131.100 (17)
Se2^iii^—U1—Pd1^vi^	133.501 (12)	Se2—Pd1—Cs1	53.905 (11)
Se1^i^—U1—Pd1^vi^	44.171 (11)	Pd2—Pd1—Cs1	102.592 (7)
Se1—U1—Pd1^vi^	44.172 (11)	U1—Pd1—Cs1	77.222 (7)
Pd1^i^—U1—Pd1^vi^	64.300 (14)	U1^v^—Pd1—Cs1	76.474 (7)
Pd1—U1—Pd1^vi^	120.745 (11)	Se1^xi^—Pd1—Cs1^v^	129.73 (2)
Pd2^iv^—U1—Pd1^vi^	120.866 (11)	Se1^v^—Pd1—Cs1^v^	53.965 (11)
Pd2—U1—Pd1^vi^	177.312 (13)	Se2^iii^—Pd1—Cs1^v^	53.904 (11)
Pd1^v^—U1—Pd1^vi^	56.446 (15)	Se2—Pd1—Cs1^v^	131.099 (17)
Se2^vii^—Cs1—Se2^viii^	80.953 (16)	Pd2—Pd1—Cs1^v^	102.592 (7)
Se2^vii^—Cs1—Se2^ix^	102.430 (16)	U1—Pd1—Cs1^v^	77.222 (7)
Se2^viii^—Cs1—Se2^ix^	160.389 (13)	U1^v^—Pd1—Cs1^v^	76.475 (7)
Se2^vii^—Cs1—Se2	160.389 (13)	Cs1—Pd1—Cs1^v^	129.363 (12)
Se2^viii^—Cs1—Se2	102.430 (16)	Se1^xi^—Pd1—Cs2^xx^	114.000 (17)
Se2^ix^—Cs1—Se2	80.953 (16)	Se1^v^—Pd1—Cs2^xx^	57.960 (16)
Se2^vii^—Cs1—Se1	94.738 (13)	Se2^iii^—Pd1—Cs2^xx^	59.002 (13)
Se2^viii^—Cs1—Se1	62.144 (11)	Se2—Pd1—Cs2^xx^	115.901 (19)
Se2^ix^—Cs1—Se1	135.669 (11)	Pd2—Pd1—Cs2^xx^	69.734 (10)
Se2—Cs1—Se1	70.690 (13)	U1—Pd1—Cs2^xx^	113.335 (9)
Se2^vii^—Cs1—Se1^ix^	62.143 (11)	U1^v^—Pd1—Cs2^xx^	111.952 (8)
Se2^viii^—Cs1—Se1^ix^	94.738 (13)	Cs1—Pd1—Cs2^xx^	158.937 (12)
Se2^ix^—Cs1—Se1^ix^	70.691 (13)	Cs1^v^—Pd1—Cs2^xx^	71.656 (7)
Se2—Cs1—Se1^ix^	135.670 (11)	Se1^xi^—Pd1—Cs2^xv^	57.960 (16)
Se1—Cs1—Se1^ix^	150.71 (2)	Se1^v^—Pd1—Cs2^xv^	114.000 (17)
Se2^vii^—Cs1—Se1^x^	70.691 (13)	Se2^iii^—Pd1—Cs2^xv^	115.903 (19)
Se2^viii^—Cs1—Se1^x^	135.670 (11)	Se2—Pd1—Cs2^xv^	59.003 (13)
Se2^ix^—Cs1—Se1^x^	62.143 (11)	Pd2—Pd1—Cs2^xv^	69.734 (10)
Se2—Cs1—Se1^x^	94.738 (13)	U1—Pd1—Cs2^xv^	113.335 (9)
Se1—Cs1—Se1^x^	86.513 (16)	U1^v^—Pd1—Cs2^xv^	111.952 (8)
Se1^ix^—Cs1—Se1^x^	100.875 (18)	Cs1—Pd1—Cs2^xv^	71.656 (7)
Se2^vii^—Cs1—Se1^xi^	135.670 (11)	Cs1^v^—Pd1—Cs2^xv^	158.937 (12)
Se2^viii^—Cs1—Se1^xi^	70.691 (13)	Cs2^xx^—Pd1—Cs2^xv^	87.301 (15)
Se2^ix^—Cs1—Se1^xi^	94.738 (13)	Se2—Pd2—Se2^xxi^	171.45 (3)
Se2—Cs1—Se1^xi^	62.144 (11)	Se2—Pd2—Se2^iii^	88.16 (2)
Se1—Cs1—Se1^xi^	100.876 (18)	Se2^xxi^—Pd2—Se2^iii^	91.20 (2)
Se1^ix^—Cs1—Se1^xi^	86.514 (16)	Se2—Pd2—Se2^xviii^	91.20 (2)
Se1^x^—Cs1—Se1^xi^	150.71 (2)	Se2^xxi^—Pd2—Se2^xviii^	88.16 (2)
Se2^vii^—Cs1—Pd1	152.457 (10)	Se2^iii^—Pd2—Se2^xviii^	171.45 (3)
Se2^viii^—Cs1—Pd1	71.604 (10)	Se2—Pd2—Pd1^xviii^	125.177 (18)
Se2^ix^—Cs1—Pd1	104.229 (10)	Se2^xxi^—Pd2—Pd1^xviii^	49.289 (12)
Se2—Cs1—Pd1	35.226 (10)	Se2^iii^—Pd2—Pd1^xviii^	125.176 (18)
Se1—Cs1—Pd1	70.632 (11)	Se2^xviii^—Pd2—Pd1^xviii^	49.289 (12)
Se1^ix^—Cs1—Pd1	121.430 (10)	Se2—Pd2—Pd1	49.289 (12)
Se1^x^—Cs1—Pd1	129.052 (12)	Se2^xxi^—Pd2—Pd1	125.177 (18)
Se1^xi^—Cs1—Pd1	34.930 (10)	Se2^iii^—Pd2—Pd1	49.289 (12)
Se2^vii^—Cs1—Pd1^xii^	35.225 (10)	Se2^xviii^—Pd2—Pd1	125.177 (18)
Se2^viii^—Cs1—Pd1^xii^	104.228 (10)	Pd1^xviii^—Pd2—Pd1	118.54 (2)
Se2^ix^—Cs1—Pd1^xii^	71.605 (10)	Se2—Pd2—U1^iv^	130.638 (16)
Se2—Cs1—Pd1^xii^	152.458 (10)	Se2^xxi^—Pd2—U1^iv^	54.919 (11)
Se1—Cs1—Pd1^xii^	129.051 (12)	Se2^iii^—Pd2—U1^iv^	130.639 (16)
Se1^ix^—Cs1—Pd1^xii^	34.930 (10)	Se2^xviii^—Pd2—U1^iv^	54.919 (11)
Se1^x^—Cs1—Pd1^xii^	70.633 (11)	Pd1^xviii^—Pd2—U1^iv^	61.640 (9)
Se1^xi^—Cs1—Pd1^xii^	121.430 (10)	Pd1—Pd2—U1^iv^	179.818 (19)
Pd1—Cs1—Pd1^xii^	156.358 (11)	Se2—Pd2—U1	54.919 (11)
Se2^vii^—Cs1—Pd1^v^	104.228 (10)	Se2^xxi^—Pd2—U1	130.639 (16)
Se2^viii^—Cs1—Pd1^v^	35.225 (10)	Se2^iii^—Pd2—U1	54.919 (11)
Se2^ix^—Cs1—Pd1^v^	152.458 (10)	Se2^xviii^—Pd2—U1	130.639 (16)
Se2—Cs1—Pd1^v^	71.605 (10)	Pd1^xviii^—Pd2—U1	179.818 (19)
Se1—Cs1—Pd1^v^	34.931 (10)	Pd1—Pd2—U1	61.639 (9)
Se1^ix^—Cs1—Pd1^v^	129.051 (12)	U1^iv^—Pd2—U1	118.178 (18)
Se1^x^—Cs1—Pd1^v^	121.430 (10)	Se2—Pd2—Cs2^xv^	58.042 (13)
Se1^xi^—Cs1—Pd1^v^	70.632 (11)	Se2^xxi^—Pd2—Cs2^xv^	114.859 (18)
Pd1—Cs1—Pd1^v^	50.637 (12)	Se2^iii^—Pd2—Cs2^xv^	114.859 (18)
Pd1^xii^—Cs1—Pd1^v^	135.902 (12)	Se2^xviii^—Pd2—Cs2^xv^	58.041 (13)
Se2^vii^—Cs1—Pd1^ix^	71.605 (10)	Pd1^xviii^—Pd2—Cs2^xv^	68.073 (10)
Se2^viii^—Cs1—Pd1^ix^	152.458 (10)	Pd1—Pd2—Cs2^xv^	68.073 (10)
Se2^ix^—Cs1—Pd1^ix^	35.225 (10)	U1^iv^—Pd2—Cs2^xv^	112.050 (5)
Se2—Cs1—Pd1^ix^	104.228 (10)	U1—Pd2—Cs2^xv^	112.050 (5)
Se1—Cs1—Pd1^ix^	121.430 (10)	Se2—Pd2—Cs2^xx^	114.860 (18)
Se1^ix^—Cs1—Pd1^ix^	70.632 (11)	Se2^xxi^—Pd2—Cs2^xx^	58.041 (13)
Se1^x^—Cs1—Pd1^ix^	34.930 (10)	Se2^iii^—Pd2—Cs2^xx^	58.041 (13)
Se1^xi^—Cs1—Pd1^ix^	129.051 (12)	Se2^xviii^—Pd2—Cs2^xx^	114.859 (18)
Pd1—Cs1—Pd1^ix^	135.903 (12)	Pd1^xviii^—Pd2—Cs2^xx^	68.073 (10)
Pd1^xii^—Cs1—Pd1^ix^	50.638 (12)	Pd1—Pd2—Cs2^xx^	68.073 (10)
Pd1^v^—Cs1—Pd1^ix^	156.358 (11)	U1^iv^—Pd2—Cs2^xx^	112.050 (5)
Se1^x^—Cs2—Se1^xiii^	136.13 (3)	U1—Pd2—Cs2^xx^	112.050 (5)
Se1^x^—Cs2—Se2^ix^	56.056 (8)	Cs2^xv^—Pd2—Cs2^xx^	86.091 (16)
Se1^xiii^—Cs2—Se2^ix^	106.862 (13)	Pd1^vi^—Se1—Pd1^v^	84.26 (3)
Se1^x^—Cs2—Se2^xiv^	106.862 (13)	Pd1^vi^—Se1—U1	81.29 (2)
Se1^xiii^—Cs2—Se2^xiv^	56.056 (8)	Pd1^v^—Se1—U1	81.29 (2)
Se2^ix^—Cs2—Se2^xiv^	137.91 (2)	Pd1^vi^—Se1—Cs1	175.16 (2)
Se1^x^—Cs2—Se2^xv^	106.862 (13)	Pd1^v^—Se1—Cs1	91.105 (9)
Se1^xiii^—Cs2—Se2^xv^	56.056 (8)	U1—Se1—Cs1	99.446 (14)
Se2^ix^—Cs2—Se2^xv^	54.431 (13)	Pd1^vi^—Se1—Cs1^x^	91.105 (9)
Se2^xiv^—Cs2—Se2^xv^	108.889 (16)	Pd1^v^—Se1—Cs1^x^	175.16 (2)
Se1^x^—Cs2—Se2^xvi^	56.056 (8)	U1—Se1—Cs1^x^	99.446 (14)
Se1^xiii^—Cs2—Se2^xvi^	106.862 (13)	Cs1—Se1—Cs1^x^	93.487 (16)
Se2^ix^—Cs2—Se2^xvi^	108.889 (16)	Pd1^vi^—Se1—Cs2^x^	88.67 (2)
Se2^xiv^—Cs2—Se2^xvi^	54.431 (13)	Pd1^v^—Se1—Cs2^x^	88.67 (2)
Se2^xv^—Cs2—Se2^xvi^	137.91 (2)	U1—Se1—Cs2^x^	166.43 (3)
Se1^x^—Cs2—Se2	82.483 (12)	Cs1—Se1—Cs2^x^	89.806 (14)
Se1^xiii^—Cs2—Se2	133.760 (13)	Cs1^x^—Se1—Cs2^x^	89.806 (14)
Se2^ix^—Cs2—Se2	70.625 (13)	Pd2—Se2—Pd1	82.01 (2)
Se2^xiv^—Cs2—Se2	150.562 (16)	Pd2—Se2—U1	80.043 (17)
Se2^xv^—Cs2—Se2	94.230 (11)	Pd1—Se2—U1	79.542 (17)
Se2^xvi^—Cs2—Se2	117.736 (13)	Pd2—Se2—Cs1	172.72 (2)
Se1^x^—Cs2—Se2^xvii^	133.761 (13)	Pd1—Se2—Cs1	90.869 (15)
Se1^xiii^—Cs2—Se2^xvii^	82.482 (12)	U1—Se2—Cs1	100.224 (13)
Se2^ix^—Cs2—Se2^xvii^	150.563 (16)	Pd2—Se2—Cs2^xv^	89.066 (18)
Se2^xiv^—Cs2—Se2^xvii^	70.624 (13)	Pd1—Se2—Cs2^xv^	87.383 (16)
Se2^xv^—Cs2—Se2^xvii^	117.735 (13)	U1—Se2—Cs2^xv^	163.979 (19)
Se2^xvi^—Cs2—Se2^xvii^	94.230 (11)	Cs1—Se2—Cs2^xv^	89.104 (12)
Se2—Cs2—Se2^xvii^	82.710 (18)	Pd2—Se2—Cs2	99.726 (15)
Se1^x^—Cs2—Se2^ii^	82.482 (12)	Pd1—Se2—Cs2	172.903 (19)
Se1^xiii^—Cs2—Se2^ii^	133.761 (13)	U1—Se2—Cs2	107.516 (12)
Se2^ix^—Cs2—Se2^ii^	117.735 (13)	Cs1—Se2—Cs2	87.161 (12)
Se2^xiv^—Cs2—Se2^ii^	94.229 (11)	Cs2^xv^—Se2—Cs2	85.771 (11)

Symmetry codes: (i) −*x*, −*y*, *z*; (ii) −*x*, *y*, *z*; (iii) *x*, −*y*, *z*; (iv) −*x*, −*y*, −*z*; (v) −*x*+1/2, −*y*, −*z*+1/2; (vi) *x*−1/2, *y*, −*z*+1/2; (vii) *x*, −*y*+1/2, −*z*+1/2; (viii) −*x*+1/2, *y*, −*z*+1/2; (ix) −*x*+1/2, −*y*+1/2, *z*; (x) −*x*, −*y*+1/2, −*z*+1/2; (xi) *x*+1/2, *y*, −*z*+1/2; (xii) *x*, *y*+1/2, −*z*+1/2; (xiii) −*x*, −*y*+1/2, *z*−1/2; (xiv) *x*−1/2, −*y*+1/2, −*z*; (xv) −*x*+1/2, −*y*+1/2, −*z*; (xvi) *x*−1/2, −*y*+1/2, *z*; (xvii) −*x*, *y*, −*z*; (xviii) *x*, *y*, −*z*; (xix) *x*−1/2, *y*+1/2, *z*; (xx) *x*+1/2, *y*−1/2, *z*; (xxi) *x*, −*y*, −*z*.
